# Evaluation of the American-English Quality of Life in Short Stature Youth (QoLISSY) questionnaire in the United States

**DOI:** 10.1186/s12955-015-0236-2

**Published:** 2015-04-02

**Authors:** Monika Bullinger, Rachel Sommer, Andreas Pleil, Nelly Mauras, Judith Ross, Ron Newfield, Lawrence Silverman, Anja Rohenkohl, Janet Fox, Julia Quitmann

**Affiliations:** Department of Medical Psychology, Center for Psychosocial Medicine, University Medical Center Hamburg-Eppendorf, Hamburg, Germany; Pfizer Inc., Outcomes Research, San Diego, CA USA; Nemours Children’s Clinic, Jacksonville, FL USA; Thomas Jefferson University, Philadelphia, USA; Rady Children’s Hospital, San Diego, CA USA; University of San Diego, San Diego, CA USA; Goryeb Children’s Hospital, Atlantic Health System, Morristown, New Jersey USA

**Keywords:** Short stature, Health related quality of life, Children, Questionnaire development, Psychometric properties

## Abstract

**Background:**

The European Quality of Life in Short Stature Youth (QoLISSY) is a novel condition-specific instrument developed to assess health related quality of life (HrQoL) in children/adolescents with short stature from patient and parent perspectives. Study objective was to linguistically validate and psychometrically test the American-English version of the QoLISSY instrument.

**Methods:**

Upon conversion of the British-English version to American-English, content validity and acceptance of the questionnaire were examined through focus group discussions with cognitive debriefing in 28 children/adolescents with growth hormone deficiency (GHD) or idiopathic short stature (ISS) and their parents. In the subsequent field test with 51 families and a re-test with 25 families the psychometric performance of the American-English version was examined and compared with the original European dataset.

**Results:**

Pilot test results supported the suitability of the American-English version. Good internal consistency with Cronbach’s Alpha ranging from 0.84 to 0.97 and high test-re-test reliabilities were observed in the field test. The QoLISSY was able to detect significant differences according to the degree of short stature with higher HrQoL for taller children. Correlations with a generic HrQoL tool support the QoLISSY’s concurrent validity. The scale’s operating characteristics were comparable to the original European data.

**Conclusion:**

Results support that the QoLISSY American-English version is a psychometrically sound short stature-specific instrument to assess the patient- and parent- perceived impact of short stature. The QoLISSY instrument is fit for use in clinical studies and health services research in the American-English speaking population.

## Introduction

Health-related Quality of Life (HrQoL) is an important multidimensional concept that is often measured in pediatric patients and instruments to measure this are increasingly being developed for that purpose. A literature review by Brutt, A.L., Sandberg, D.E., Chaplin, J., Wollmann, H., Noeker, M., Koltowska-Haggstrom, M. and Bullinger, M. [1] identified five pediatric condition-specific instruments available for use in children and adolescents with short stature. According to the authors, none of these measures provides adequate assessment of all domains potentially relevant to the condition. Therefore, the purpose of the European QoLISSY (Quality of Life in Short Stature Youth) effort was to develop cross-culturally and psychometrically test a questionnaire that utilizes a multifactorial approach to evaluate the impact of short stature on children and adolescents, from both the child and parent perspectives.

The original QoLISSY questionnaire was developed along a common study protocol in five European countries and respective languages (UK, Spain, France, Sweden and Germany). Clinically referred children and adolescents diagnosed with growth hormone deficiency (GHD) or idiopathic short stature (ISS) and their parents were included in the study. To make the QoLISSY questionnaire available for American-English speaking children and adolescents with short stature living in the US, it is necessary to examine the instrument both in terms of conceptual equivalence as well as psychometric performance. The aim of the present study is the translation and psychometric evaluation of the QoLISSY instrument for use in the US.

### Health-related quality of life (HrQoL) in short stature

Health-related Quality of Life reflects the impact of chronic diseases and disabilities on wellbeing and functioning of patients and their families. Assessments of HrQoL can help to increase our understanding of the burden of disease, examine treatment outcomes, evaluate quality of care and assist in tailoring interventions according to patients’ needs [2].

Generic HrQoL assessment makes it possible to compare HrQoL along the health continuum and across different health conditions [3]. Generic measures, however, may not be sensitive enough to detect smaller changes in HrQoL and may not cover all relevant domains for the respective disorder. Targeted HrQoL instruments focus on the impact of the condition; hence offering a deeper insight into the HrQoL of patients with a specific impairment, condition, or disorder. But, since those instruments are specific to a defined health condition, their use for comparisons between different conditions is limited [3].

Health-related Quality of Life can be measured either by patient-report or by observer-report of a significant other such as a parent, other caregiver or clinician. Parent reports, however, do not necessarily reflect the child’s entire perspective, especially when evaluating the children’s feelings such as anxieties, or behaviors such as interactions with peers [4]. Studies suggest that parents of healthy children tend to overestimate, while parents of ill children underestimate their children’s quality of life but the direction of divergence can vary across chronic conditions [5,6].

Instruments to assess HrQoL exist for different age groups, but younger children are underrepresented [7]. Only one instrument (TACQOL-S) [8] identified in the review by Brutt, A.L., Sandberg, D.E., Chaplin, J., Wollmann, H., Koltowsa-Haggestrom, M., Bullinger, M. [1] is available in three languages, which makes cross-cultural comparisons difficult [9]. To gain more insight into the HrQoL of children, especially those with short stature, the development of targeted instruments is necessary, such as the QoLISSY questionnaire, to assess their HrQoL [10].

### Short stature

Short stature is commonly defined as a height 2.0 or more standard deviations (*SD*) below the population-specific mean height for age and gender. Endocrine and non-endocrine causes for short stature have been identified, including growth hormone (GH) deficiency (GHD), which can occur both alone or in combination with other pituitary hormone deficits and is treated by GH replacement [11]. However, about 50% of children fulfilling the statistical definition of short stature, have no identifiable underlying endocrine or other disorder and are designated as Idiopathic Short Stature (ISS) [12]. These children are born with normal length and weight (> − 2.0 *SD*), have low growth velocity but show no evidence of hypothyroidism, malnutrition or lack of GH [13]. Treatment with GH in children with ISS may result in an increase in height but the responses are more variable than in GHD [14].

### Psychosocial consequences of short stature

Height has been found to be a factor affecting the HrQoL of adults on a population level [15]; however, similar epidemiological studies in children are lacking. In the case of health-referred children with short stature, studies have identified socio-emotional problems. In a study from Voss and Mulligan [15] for example, short children reported that they were bullied frequently [16]. Short stature has also been reported to be associated with stigmatization and social isolation which can cause chronic psychosocial stress [17]. Though these experiences do not necessarily result in clinically relevant problems, parents report their children to be less socially competent and to generally have more problems of a social nature than parents of children with normal height [18]. Children with GHD or ISS have also been shown to exhibit social and physical functioning limitations as assessed with the Child Behavior Check List [19].

A German study compared short statured children with a group of children of normal height using the KIDSCREEN 52, a generic self-report instrument to assess quality of life [20]. Short statured children scored significantly lower on most scales, e.g. Psychological Well-being, Self-Perception and Social Support & Peers, but did not significantly differ from the non-short children on the Parent Relations & Home Life and Financial Resources scales [17].

Other studies examined if growth hormone treatment increases wellbeing and functioning. Abe et al. [20] reported that depressive mood in children with GHD improved with growth hormone treatment. Chaplin et al. [21] reported significant treatment benefits regarding behavioral problems and depressive mood with GH. Zlotkin and Varma [22] reviewed four studies and concluded that there is an increase in wellbeing in children receiving growth hormone treatment, especially in those diagnosed with GHD [23].

Visser-van Balen et al. [23] reported a height gain of 2.3 cm compared to a control group for ISS and Small for Gestational Age (SGA) patients, but no differences in self-reported height-related psychosocial problems. Interestingly, most of the subjects, treated and untreated, were happy with their achieved height [24]. Sandberg and Colsman suggested that psychosocial problems in children with short stature are common phenomena and do not necessarily result from short stature [25].

Health-related Quality of Life instruments have rarely been used in comparative trials, so that information on HrQoL in short statured children as an indicator of treatment efficacy, particularly in the context of clinical efficacy trials of GH treatment, is missing. One reason for this is unavailability of an appropriate measure to assess HrQoL across languages and cultures. The condition-specific QoLISSY questionnaire was developed to fill this gap [9]. The current study describes the adaptation of the QoLISSY to American-English and examines its operating characteristics and psychometric properties.

## Methods

Within the cultural adaptation, the original British-English QoLISSY version was modified by independent native American-English speakers. The American-English pilot version resulted in nominal wording changes in the response categories only. These were: ‘Almost never’ (US) was used instead of ‘Seldom’ (UK) as well as ‘Sometimes’ (US) instead of ‘Quite often’ (UK) and ‘Almost always’ (US) instead of ‘Very often’ (UK). Procedures originally applied within the European QoLISSY project were also used in the current validation process [9], which was performed in cooperation with four pediatric endocrine clinics in the US. In the first phase of the study, patients and parents participated in separate moderator-supported focus group sessions/discussions. Afterwards, these patients and parents separately completed the QoLISSY questionnaire and subsequently participated in a cognitive debriefing exercise. During the debriefing, they were specifically asked to evaluate the items in terms of clarity, sensitivity, importance, and relevance for their personal situation. The transcripts of the discussions were analyzed and questionnaires were modified according to the results. The resulting American-English QoLISSY questionnaire was used in the second phase of the study, in which a field test was conducted in children with GHD or ISS and their parents who were recruited from the participating clinical centers. Questionnaires were distributed to participating families and returned to the sites. A second set of questionnaires was mailed to the participants approximately two weeks later to provide a re-test reliability dataset.

### Inclusion and exclusion criteria

Short statured children between eight and 18 years of age and one or both of their parents were invited to participate in the validation study. In addition, the parents of younger children (those between four and seven years) were included. All children had a clinical diagnosis of either ISS or GHD. Children with multiple hormone deficiencies or severe mental or physical conditions were excluded. Children could be either untreated or treated with GH for varying durations. The study was approved by each center’s institutional review committee and parents signed an informed consent before participation, while children and adolescents additionally signed an assent form if required.

### Questionnaires

The QoLISSY instrument consists of three core scales which cover *Physical* (six items), *Social* (eight items) and *Emotional* (eight items) aspects of functioning and well-being in child- and parent-report. Furthermore, to identify potential determinants of HrQoL three additional scales that cover aspects of Coping (ten items), general Beliefs about height (five items) and Treatment specific aspects (for GH-treated children and their parents only, 14 items). Two additional scales apply to and are completed by parents only. They contain questions about the child’s future (four items) and Effects of their child’s short stature on parents (eleven items). Based on scoring rules of standard HrQoL measures [26] and in line with the European QoLISSY manual, the QoLISSY Total Score is calculated as a mean of the three core module means. If 80% or more of all items in a scale are completed by the respondent, the scale score mean was calculated as the mean of the endorsed items, otherwise the scale score and the resulting Total Score were treated as missing.

The KIDSCREEN Index questionnaire measuring HrQoL from both patients and parents perspective was included as a generic measure to examine convergent validity of the QoLISSY for psychometric testing purposes as a reliable and valid generic instrument [27]. Socio-demographic and clinical data were collected by the clinical centers including age, gender, diagnosis, height, treatment status, duration of treatment, date of treatment start.

### Analysis

Data collected from the focus groups were processed using MaxQDA, a qualitative data analysis program (VERBI-Software MaxQDA 10). Parallel coding involving two trained coding experts was used. For each statement inter-observer agreement was discussed and the few disagreements in statement codings were resolved by consensus. The purpose of the field test and re-test, which followed, was to evaluate the operating characteristics for both the children’s and parents’ version of the American-English questionnaire. Item distribution characteristics such as mean (*M*), standard deviation (*SD*), percentage of items at the lower (floor effects) and the higher end (ceiling effects), skewness as well as discriminatory power of the scales were inspected. Two indices for internal consistency were measured: split-half reliability and Cronbach’s alpha. Scores above 0.70 were interpreted as acceptable [28]. Test re-test reliability was calculated with the intra-class correlation coefficient (ICC, *r*) for each scale and the total score in both the parents’ and the children’s version. Intra Class coefficients between 0.40 and 0.75 indicate fair to good reliability and above 0.75 excellent reliability [29].

To test for convergent validity, the QoLISSY scale scores were correlated with the KIDSCREEN Index in the patient-report and the parent report version (Pearson’s Correlation Coefficient). In line with Weber J and Lamb D [30] scores between r = 0.36 and r = 0.67 were interpreted as moderate. Scores between r = 0.68 to r = 0.90 indicate high correlations and scores above 0.90 indicate very high correlations. In line with published research suggesting effects of clinical characteristics on HrQoL, known-groups validity was tested based on the degree of short stature (≤ −2.0*SD*, > − 2.0*SD*). Differences in treatment status were not analyzed due to the small number of untreated children/ adolescents.

Finally, differences according to patients’ gender and age were evaluated in order to examine the need for respective adjustments in scoring. Convergence of operating characteristics between the European and the US samples was inspected albeit limited in that the samples, though similar, were not necessarily equivalent.

## Results

### Focus groups

To ensure that this study used a method similar to the one used in the original European study, a focus group and cognitive debriefing manual, including interview guidelines, was prepared. Bi-lingual trained moderators (psychologists, medical staff) from Europe and the US conducted initial interviews. The focus group discussions and interviews were transcribed verbatim and analyzed by two independent raters, using the qualitative content analysis program MAX QDA [31]. The detailed results of the qualitative analyses will be published in a subsequent paper.

Focus groups with cognitive debriefing included 24 children and adolescents and 28 parents. Overall, the qualitative analyses of the focus group responses produced no new concepts or topics, nor excluded any topics of relevance when compared with the results of the original European focus groups. The cognitive debriefing questions for children/adolescents resulted only in nominal wording changes. For example: ‘I am concerned that (s)he will be hurt by any insults about his/ her height’ (US) was used instead of ‘Because of my child’s height I am concerned that (s)he will be hurt by the insults’ (UK). For the focus group and cognitive debriefing phase the 5-point Likert-like response category labels were adapted to an American-English format from the commonly used British-English form of the scale point labels.

### Field-test

A total of 60 families participated on the field test with 51 sets of responses received from the parent and 50 sets of their child/adolescent and nine questionnaires from exclusively the parents of children between four to seven years of age; one boy in the age group 13–18 did not answer the QoLISSY child version in the field test. Male patients outnumbered females about 2:1 and this was consistent across age groups. In total, 28 children were diagnosed with GHD and 32 with ISS, 55 patients were treated with GH, and 5 patients were untreated (see Table 1). At the time of the field test data collection, 25 children still met the criteria for short stature with a standard deviation (*SD*) below −2.0; while 35 had grown post-treatment and were taller than −2.0 *SD*. The 51 families who had participated in the field test were asked to complete a retest questionnaire two weeks later. A total of 25 out of 51 families returned the questionnaire for this purpose.

**Table 1:** **Sample characteristics**

				**Age group**				
		**Total**		**4-7 years**	**8-12 years**		**13-18 years**	
		**Child**	**Parent**	Parent only *	**Child**	**Parent**	**Child**	**Parent**
**Gender**	**Male**	35 (68.6%)	41 (68.3%)	6 (66.7%)	12 (54.5%)	12 (54.5%)	23 (79.3%)	23 (79.3%)
	**Female**	16 (31.4%)	19 (31.7%)	3 (33.3%)	10 (45.5%)	10 (45.5%)	6 (20.6%)	6 (20.7%)
**Diagnosis**	**ISS**	26 (51.0%)	32 (53.3%)	6 (66.7%)	13 (59.1%)	13 (59.1%)	13 (42.9%)	13 (44.8%)
	**GHD**	25 (49.0%)	28 (46.7%)	3 (33.3%)	9 (40.9%)	9 (40.9%)	16 (57.1%)	16 (55.2%)
**Treatment**	**Treated**	46 (90.2%)	55 (91.7%)	9 (100%)	20 (90.9%)	20 (90.9%)	26 (89.3%)	26 (89.7%)
	**Untreated**	5 (9.8%)	5 (8.3%)	0 (0%)	2 (9.1%)	2 (9.1%)	3 (10.7%)	3 (10.3%)
***SD*** **height**	**> − 2.0**	31 (60.8%)	35 (58.3%)	4 (44.4%)	13 (59.1%)	13 (59.1%)	18 (62.1%)	18 (62.1%)
	**≤ − 2.0**	20 (39.2%)	25 (41.7%)	5 (55.5)	9 (40.9%)	9 (40.9%)	11 (37.9%)	11 (37.9%)

*Only parents completed the questionnaire for children aged 4–7.

In terms of mean (*M*), standard deviation (*SD*), range and floor and ceiling effects scale characteristics of the American-English version of the self- and parent-report show a slight skew to the right, favoring a higher QoL within the range of 0 to 100 (with 100 reflecting the highest QoL). Cronbach’s alpha measure of internal consistency across all QoLISSY scales ranged between α = 0.57 (*Coping*) to α = 0.96 (*QoL Total Score*) for the child/adolescent version (Table 2) and from α = 0.87 (*Treatment*) to α = 0.97 (*QoL Total Score*) for the parent version (Table 3). Test-re-test-reliability ranged from *r* = 0.475 (*Emotional QoL*) up to *r* = 0.815 (*Physical QoL*) for the patient reported version and from *r* = 0.549 (*Treatment and Coping*) up to *r* = 0.893 (*Physical QoL*) for the parent reported version. Satisfactory instrument reliabilities were found in the children‘s/adolescent’s (Table 2) and the parent version of the QoLISSY questionnaire (Table 3). Comparison of Cronbach’s alpha of the US with the European data set, as shown in Tables 2 and 3, suggests equivalence in the reliability of the instrument.

**Table 2:** **Descriptive statistics and reliability estimates on QoLISSY scale level for the US sample in comparison with the European sample (children)**

**Children**	**Descriptive statistics**						**Reliability**			
	***N***	**Mean**	***SD***	**Skewness**	**% Floor**	**% Ceiling**	**N**	**α**	**Split-half**	**ICC**
Physical*	49	75.54	19.89	−0.91	2.0	8.2	48	0.85	0.87	0.82
**US sample**										
European sample	268	73.69	20.08	−0.95	0.4	12.3	263	0.84	0.83	0.80
Social*	50	71.42	20.72	0.21	2.0	8.0	49	0.92	0.94	0.69
**US sample**										
European sample	268	72.94	22.93	−0.79	0.4	10.4	265	0.87	0.83	0.80
Emotional*	50	73.13	19.35	−0.84	2.0	10.0	49	0.89	0.95	0.48
**US sample**										
European sample	268	72.69	23.87	−0.94	1.1	10.1	259	0.88	0.88	0.85
**Coping**	48	50.46	20.61	0.25	2.1	2.1	45	0.57**	0.53	0.77
**US sample**										
European sample	257	55.60	22.38	−0.19	1.2	1.2	244	0.82	0.65	0.56
**Beliefs**	49	69.34	27.40	−0.75	2.0	22.4	49	0.87	0.87	0.59
**US**										
European sample	266	69.13	28.59	−0.78	3.8	21.1	263	0.85	0.85	0.83
**Treatment**	43	49.82	19.06	0.32	2.3	2.3	41	0.84	0.70	0.67
**US**										
European sample	152	55.12	21.06	−0.13	0.7	0.7	143	0.87	0.74	0.73
**QoL Total Score**	**49**	**73.75**	**21.35**	**−0.82**	**2.0**	**2.0**	**47**	**0.96**	**0.93**	**0.72**
**US sample**										
**European sample**	**268**	**73.10**	**21.39**	**−0.80**	**0.4**	**4.9**	**251**	**0.95**	**0.92**	**0.88**

*The QoL total Score is the sum of the physical, social and emotional scale.

**If item three is deleted, Cronbachs alpha would be 0.81.

**Table 3:** **Descriptive statistics and reliability estimates on QoLISSY scale level for the US sample in comparison with the European sample (parents)**

**Parents**	**Descriptive statistics**						**Reliability**			
	**N**	**Mean**	**SD**	**Skewness**	**% Floor**	**% Ceiling**	**N**	**α**	**Split-half**	**ICC**
Physical*	60	70.39	21.92	−0.50	1.7	10.0	59	0.91	0.86	0.89
**US sample**										
European sample	317	71.80	23.20	−0.76	1.3	13.2	310	0.86	0.86	0.84
Social*	60	65.92	26.02	−0.41	3.3	11.7	59	0.95	0.94	0.82
**US sample**										
European sample	313	69.41	25.24	-.60	0.3	12.5	308	0.90	0.88	0.84
Emotional*	59	68.05	22.11	−0.62	1.7	6.8	56	0.92	0.92	0.80
**US sample**										
European sample	315	68.50	22.90	−0.66	0.3	5.4	309	0.88	0.90	0.70
**Coping**	56	40.27	23.41	−0.10	10.7	1.8	54	0.91	0.84	0.55
**US sample**										
European sample	286	45.07	20.96	0.03	3.1	0.7	276	0.86	0.65	0.65
**Beliefs**	58	62.72	31.72	−0.36	1.7	20.7	58	0.94	0.94	0.78
**US sample**										
European sample	310	67.62	28.95	−0.62	2.6	22.3	310	0.90	0.89	0.72
**Treatment**	54	52.73	19.35	0.09	1.9	1.9	52	0.87	0.74	0.55
**US sample**										
European sample	163	55.18	20.60	−0.23	0.6	1.2	154	0.88	0.78	0.74
**Future ** **US sample**	59	73.73	25.40	−0.90	1.7	23.7	59	0.94	0.92	0.85
European sample	303	74.85	26.45	−0.1.1	1.3	20.5	297	0.90	0.86	0.74
**Effects on parents**	60	64.80	22.68	−0.68	3.3	1.7	59	0.90	0.85	0.71
**US sample**										
European sample	313	65.68	24.48	−0.44	1.6	5.1	261	0.90	0.82	0.88
**QoL Total Score**	**59**	**68.31**	**22.14**	**−0.50**	**1.7**	**1.7**	**54**	**0.97**	**0.94**	**0.85**
**US sample**										
European sample	**313**	**69.97**	**22.03**	**−0.65**	**0.3**	**2.9**	**296**	**0.95**	**0.91**	**0.86**

*The QoL total Score is the sum of the physical, social and emotional scale.

Construct validity was determined using convergent and known-groups validity. Significant correlations in the mean range with the KIDSCREEN Index, as an indicator of convergent validity, were found for the QoLISSY *Physical* (*r* = 0.36), *Social* (*r* = 0.33) and *Emotional* scale (*r* = 0.29) in patient self-report, but only for the QoLISSY *Emotional* scale (*r* = 0.36) in parent-report. Similar correlation coefficients, indicating less than 10% of shared variance, were found in the European data set (see Table 4).

**Table 4:** **Correlations between QoLISSY scales and KIDSCREEN Index in the US sample in comparison with the European sample**

**QoLISSY scales**	**KIDSCREEN**	**KIDSCREEN**
	**Index (patient completed)**	**Index (parent completed)**
	**N = 46**	**N = 59**
**Physical**	0.36*	0.18
**US sample**		
European sample	0.25**	0.23**
**Social**	0.33*	0.22
**US sample**		
European sample	0.27**	0.21**
**Emotional**	0.29*	0.36**
**US sample**		
European sample	0.31**	0.30**
**Coping**	−0.04	0.02
**US sample**		
European sample	0.14	0.21**
**Beliefs**	0.12	0.25
**US sample**		
European sample	0.32**	0.21**
**Treatment**	−0.02	0.16
**US sample**		
European sample	0.13	0.11
**Future US sample**	n.a.	0.29*
European sample	n.a	0.17**
**Effects on Parents**	n.a	0.24
**US sample**		
European sample	n.a	0.17**
**QoL Total Score**	0.34*	0.279*
**US sample**		
European sample	0.30**	0.27**

*Pearson Correlation Coefficient “r” is significant at the 0.05 level (2-tailed).

**Pearson Correlation Coefficient “r” is significant at the 0.01 level (2-tailed).

n.a. not applicable, since scales were completed by parents only.

Known groups validity was analyzed by comparing patient subgroups according to height (≤ −2.0 *SD* vs. > −2.0 *SD*). Differences in mean scores of each of the QoLISSY scales were analyzed separately for the patient and the parent-reported version of the questionnaire (Table 5).

**Table 5:** **Differences in QoLISSY scales according to height deviation (** ***t*** **-test) in child report and in parent report**

	**Height > −2.0** ***SD***			**Height ≤ −2.0** ***SD***					
	**(taller)**			**(shorter)**					
**Scale**	**n**	Mean*	**SD**	**n**	Mean*	**SD**	**t**	**df**	**p**
**Physical**									
Child report^1^	30	80.97	14.99	19	66.97	23.80	−2.53	47	**0.015**
Parent report	35	79.12	17.80	25	58.16	21.57	−4.11	58	**0.001**
**Social**									
Child report	30	79.27	19.77	20	59.64	28.25	−2.90	48	**0.006**
Parent report	35	75.06	22.57	25	53.13	25.50	−3.52	58	**0.001**
**Emotional**									
Child report^1^	30	79.69	15.91	20	63.28	27.99	−2.38	27.2	**0.025**
Parent report	35	72.23	19.84	24	61.94	24.18	−1.79	57	0.079
**Coping**									
Child report	28	45.06	17.45	20	58.03	42.47	1.46	46	0.152
Parent report	34	36.25	24.00	22	46.48	21.53	1.62	54	0.111
**Beliefs**									
Child report	29	73.71	23.59	20	63.13	30.27	−1.37	47	0.176
Parent report	35	66.61	30.31	23	56.79	33.55	−1.16	56	0.253
**Treatment**									
Child report	28	48.71	15.47	15	51.90	20.15	0.580	41	0.565
Parent report	33	55.98	19.83	21	47.62	17.85	−1.57	52	0.123
**Future**									
Parent report	35	78.43	23.16	24	66.88	27.42	−1.75	57	0.086
**Effects on Parents**									
Parent report	35	67.40	23.16	25	61.16	21.94	−1.05	58	0.298
**Total Score**									
Child report^1^	30	79.98	15.45	19	63.92	25.76	−2.45	26.3	0.021
Parent report	35	75.47	19.03	24	57.87	22.57	−3.23	57	0.002

The Scales “Future” and “Effects on Parents” only exist in the parents’ version.

^1^Levene’s test for variance homogeneity is significant with p < .05.

*Minimum = 0, maximum = 100; the higher the better.

In the child/adolescent reported QoLISSY, significant differences between the groups based on height were found in the following scales: *Physical* (*p* = 0.043) *Social* (*p* = 0.009) and *Emotional* (*p* = 0.044), confirming that taller children have better quality of life, as would be expected. The total score provided similar evidence of discriminant validity (*p* = 0.035).

The parent-report version analysis yielded comparable results in that group differences based on height *SD* were found for the *Physical* (*p* = 0.001), *Social* (*p* = 0.001) and the QoL Total Score (*p* = 0.003), but failed to reach significance in the scale *Emotional.* These results reflect similar findings from the European data regarding height (data not shown here) [32].

## Discussion

The translation and psychometric testing of the American-English version of the European QoLISSY questionnaire was conducted following generally accepted (FDA) guidelines for instrument validation [33]. Qualitative focus group discussions did not yield any novel differences, indicating conceptual equivalence and comparability of the short stature experience between the American-English and the European sample. Cognitive debriefing results support that the instrument is acceptable and understandable to children/adolescents and parents in the US. The pilot test results were sufficiently robust to allow for the larger field test without the need for additional changes to the instrument. The field test data show results that were psychometrically satisfactory, including good operating characteristics, sufficient evidence of reliability, and acceptable evidence of construct validity in the child-report. The lowest ICCs we observed were between 0.48 and 0.69 in child-report and 0.55 and 0.71 in parent-report. According to Rosner, B. [29] these can be considered as fair to good. The poor internal consistency of the child-reported Coping scale is due to one item which may have been misunderstood by the respondents. We decided to keep rather than omit this item, in order to ensure the comparability with the appropriately performing parent-report and the European data set. A modification of the item for future studies will be considered. Significant positive correlations between the three QoLISSY core scales and the KIDSCREEN Index suggest convergent validity, however, with a percentage of shared variance below 10% for child-reported values. For the parent-report, no evidence of convergent validity could be found. These results support the need for the development of a condition-specific tool such as the QoLISSY questionnaire. Significant group differences in the scales for both the child and parent cohorts, based on the degree of short stature, are evidence of discriminant validity. The scales *Physical*, *Social* and *Emotional* confirm that taller children have better quality of life, as would be expected. The total score provided similar evidence of discriminant validity. Comparing distributional characteristics of the American-English sample with the European sample supports the equivalence of the measure in the two populations. Additionally, inspection of coefficients for reliability (internal consistency and test-re-test) as well as validity (convergent and known groups) supports comparability of psychometric performance of the QoLISSY in the American-English and European samples. These findings further underscore the results of the cognitive debriefing and suggest that the conceptual framework of short stature is consistent across the western cultures of Europe and the US. How this translates into other geographies and cultures has yet to be evaluated.

### Limitations

The sample size in this study was too small to conduct a confirmatory factor analysis, but sufficient for the purposes of descriptive comparisons of psychometric characteristics between the US and the European QoLISSY versions. Future studies with sufficient sample sizes are needed to conduct a confirmatory factor analysis for the US QoLISSY version. Three geographical regions in the US were represented, but only English speaking subjects were included in the current analysis. The American-Spanish version of the QoLISSY, specifically one that considers the possible cultural differences of a Spanish-American population, may be necessary if one would apply this measure to this large segment of the US population. In addition, while appropriately weighted in terms of diagnosis and current height above and below −2.0 *SD*, the US sample included only a few GH-untreated patients, which affected the detection of treatment-related group differences. Finally, the Qo-LISSY score distribution in terms of standard deviation and range as well as the scale effect size were comparable across Europe and the US, as was the instrument’s ability to differentiate between groups based on height. Because of differences in recruitment and sample composition, these differences cannot be interpreted to indicate a comparable quality of life experience by patient- and parent-observer report. More than scale-based psychometric criteria, known group validity is dependent on sample composition. Therefore, additional studies in large and comparable samples are necessary to further evaluate the factor structure of the US QoLISSY and the comparability of the measure across countries.

## Conclusion

Results of pilot testing with focus group discussions and cognitive debriefing show that HrQoL concepts are adequately represented for the US population. Inspection of the psychometric test criteria in terms of reliability and validity indicate comparable performance of the measure in the US and in Europe.

The cross-culturally applicable QoLISSY provides a step forward in allowing measurement of the HrQoL impact of short stature in children and adolescents and their parents. It is now available for patients and parents in the US. The instrument is fit for use to measure outcomes in clinical trials and may be useful as well to assess treatment outcomes in a clinical practice environment. Further refinement, additional validation in other languages and cultures, migration to electronic platforms and development of a shorter version of the QoLISSY questionnaire are under consideration, to allow a broader use of the measure in children and adolescents with short stature from their own and from their parents’ perspective.

### Access to the QoLISSY instrument

QoLISSY is a joint initiative between Pfizer Limited and the University Medical Center Hamburg - Eppendorf. Copyright Pfizer Limited all rights reserved. The European QoLISSY instrument, together with comprehensive information of its development and validation process is published in the QoLISSY’s User’s Manual (Pabst Science Publishers, Lengerich, 2013). The Manual, which is available upon request, includes QoLISSY child and parent forms, as well as scoring information.
